# Antibacterial Activity and Synergistic Antibacterial Potential of Biosynthesized Silver Nanoparticles against Foodborne Pathogenic Bacteria along with its Anticandidal and Antioxidant Effects

**DOI:** 10.3389/fmicb.2017.00167

**Published:** 2017-02-15

**Authors:** Jayanta Kumar Patra, Kwang-Hyun Baek

**Affiliations:** ^1^Research Institute of Biotechnology and Medical Converged Science, Dongguk University-SeoulGoyang-si, South Korea; ^2^Department of Biotechnology, Yeungnam UniversityGyeongsan, South Korea

**Keywords:** antibacterial, anticandidal, antioxidant, foodborne bacteria, green synthesis, silver nanoparticles, *Zea mays*

## Abstract

Silver nanoparticles plays a vital role in the development of new antimicrobial substances against a number of pathogenic microorganisms. These nanoparticles due to their smaller size could be very effective as they can improve the antibacterial activity through lysis of bacterial cell wall. Green synthesis of metal nanoparticles using various plants and plant products has recently been successfully accomplished. However, few studies have investigated the use of industrial waste materials in nanoparticle synthesis. In the present investigation, synthesis of silver nanoparticles (AgNPs) was attempted using the aqueous extract of corn leaf waste of *Zea mays*, which is a waste material from the corn industry. The synthesized AgNPs were evaluated for their antibacterial activity against foodborne pathogenic bacteria (*Bacillus cereus* ATCC 13061, *Listeria monocytogenes* ATCC 19115, *Staphylococcus aureus* ATCC 49444, *Escherichia coli* ATCC 43890, and *Salmonella* Typhimurium ATCC 43174) along with the study of its synergistic antibacterial activity. The anticandidal activity of AgNPs were evaluated against *Candida* species (*C. albicans* KACC 30003 and KACC 30062, *C. glabrata* KBNO6P00368, *C. geochares* KACC 30061, and *C. saitoana* KACC 41238), together with the antioxidant potential. The biosynthesized AgNPs were characterized by UV-Vis spectrophotometry with surface plasmon resonance at 450 nm followed by the analysis using scanning electron microscope, X-ray diffraction, Fourier-transform infrared spectroscopy and thermogravimetric analysis. The AgNPs displayed moderate antibacterial activity (9.26–11.57 mm inhibition zone) against all five foodborne pathogenic bacteria. When AgNPs were mixed with standard antibacterial or anticandidal agent, they displayed strong synergistic antibacterial (10.62–12.80 mm inhibition zones) and anticandidal activity (11.43–14.33 mm inhibition zones). In addition, the AgNPs exhibited strong antioxidant potential. The overall results highlighted the potential use of maize industrial waste materials in the synthesis of AgNPs and their utilization in various applications particularly as antibacterial substance in food packaging, food preservation to protect against various dreadful foodborne pathogenic bacteria together with its biomedical, pharmaceutical based activities.

## Introduction

Nanotechnology is an emerging field of interdisciplinary research that includes all spheres of science starting from physics, chemistry, biology, and especially biotechnology (Natarajan et al., 2010). Nanoparticles (NPs) are a group of materials synthesized from a number of metals or non-metal elements with distinct features and extensive applications in different fields of science and medicine (Matei et al., 2008). Among them, silver nanoparticles (AgNPs) have been extensively studied because of their good electrical conductivity, as well as their potential for use in optical applications in nonlinear optics, as spectrally selective coatings for solar energy absorption, biolabeling, intercalation materials for electrical batteries as optical receptors, and catalysts in chemical reactions. Nanoparticles also have potential biological applications, such as biosensing, catalysis, drug delivery, imaging, nano device fabrication, and for use as antimicrobial agents and in medicine (Ghosh et al., 1996; Geddes et al., 2003; Nair and Laurencin, 2007; Jain et al., 2008; Sharma et al., 2009; Zargar et al., 2014). AgNPs release Ag^+^ ions that interact with the thiol groups in bacterial proteins and affect the DNA replication, resulting in destruction of the bacteria (Marini et al., 2007). Additionally, nanoparticles have been shown to have potential anti-bacterial activity and significantly higher synergistic effects when applied with many antibiotics (Devi and Joshi, 2012).

Synthesis of AgNPs employing chemical and physical methods has been extensively studied throughout the world; however, these methods are often environmentally toxic, technically laborious and economically expensive (Gopinath et al., 2012). Accordingly, biological methods for synthesis of AgNPs using plants, microorganisms and enzymes have been suggested as possible eco-friendly alternatives (Mohanpuria et al., 2008). The synthesis of AgNPs using plants or plant extracts as reducing and capping agents is considered advantageous over other biological processes because they eliminate the need for the elaborate process of culturing and maintaining biological cells, and can be scaled up for large-scale nanoparticle synthesis (Saxena et al., 2012; Valli and Vaseeharan, 2012). Overall, plant-mediated nanoparticles synthesis is a cost-effective, environmentally friendly, a single-step method for biosynthesis process that is safe for various human therapeutic and food based uses (Kumar and Yadav, 2009). Generally, the AgNO_3_ is reduced by the action of the reducing agents (plant extracts) to form silver nanoparticles which are further stabilized by the bioactive compounds from the biological extracts to form a stable silver nanoparticle.

During recent years, the use of agricultural and industrial wastes in the synthesis of different types of metal nanoparticles has been extensively investigated (Basavegowda and Lee, 2013; Ramamurthy et al., 2013; Nezamdoost et al., 2014) A number of food crops are industrially used for production of different types of food products and processed food. Among these, maize (*Zea mays*) is widely used throughout the world for production of popcorn, chips, corn oil, corn starch, and many other materials. Only the kernels of the corn plant are edible, while rest of the crop are occasionally used as animal feed or ingredients in beverages. Different parts of the maize plant have been effectively utilized in traditional medicines as strong therapeutic agents (Konstantopoulou et al., 2004; Ullah et al., 2010; Solihah et al., 2012). A number of bioactive compounds, such are polyphenols (chlorogenic acid, caffeic acid, rutin, ferulic acid, morin, quercetin, naringenin, and kaempferol), anthocyanins, flavonoids, flavonols, and flavanols have been reported to be present in the *Z. mays* plant and its various parts such kernel, leaves, roots etc., which are responsible for its antioxidant, antiinflammatory and other medicinal potential (Ramos-Escudero et al., 2012; Bacchetti et al., 2013; Pandey et al., 2013). Hence utilization of maize waste materials in the synthesis of nanoparticles would be a profitable approach in ecofriendly and cost effective nanoparticle synthesis.

Recently there is emergence of multi drug resistant pathogenic bacterial strains and most of the available antibiotics are not active against these pathogens (Andersson and Hughes, 2010; Huh and Kwon, 2011). These drug resistant pathogens are more pathogenic with high mortality rate than that of wild strain. The scientific community is continuously searching for a new classes of disinfection systems that could act efficiently against these pathogens. Silver-containing systems, and especially the AgNPs are these days one of the strong alternatives in search for various antibacterial drugs, as these nanoparticles have been reported previously to exhibit interesting antibacterial activities against a broad spectrum of pathogenic bacteria (Shahverdi et al., 2007; Lara et al., 2011; Guzman et al., 2012; Rai et al., 2012; Ouay and Stellacci, 2015). However, studies on AgNPs are still under investigation as antimicrobial and the studies performed since now demonstrated that a case by case evaluation have to be done for each nanoparticle and bacterial target. The bactericidal effects of ionic silver and the antimicrobial activity of colloidal silver particles is generally influenced by the size of the particles, i.e., the smaller the particle size, the greater the antimicrobial activity (Zhang et al., 2003).

There are many advantages of AgNPs to be used as an effective antimicrobial agents. They are highly effective against a broad range of microbes and parasites, even at a very low concentration with very little systemic toxicity toward humans (Ouay and Stellacci, 2015). AgNPs have been reported to be used and tested for several applications including prevention of bacterial colonization and elimination of microorganisms on various medical devices, disinfection in wastewater treatment plants, and silicone rubber gaskets to protect and transport food and textile fabrics (Guzman et al., 2012).

The present study investigated synthesis of AgNPs using the waste leaves of ears of corn following a green route and evaluate its potential application as antibacterial compound against a number of five foodborne pathogenic bacteria (*Bacillus cereus* ATCC 13061, *Listeria monocytogenes* ATCC 19115, *Staphylococcus aureus* ATCC 49444, *Escherichia coli* ATCC 43890, and *Salmonella* Typhimurium ATCC 43174) along with its anticandidal potential against five different *Candida* species (*C. albicans* KACC 30003 and KACC 30062, *C. glabrata* KBNO6P00368, *C. geochares* KACC 30061, and *C. saitoana* KACC 41238) and their antioxidant potentials. Utilization of these industrial waste materials in the synthesis of nanoparticles could add the values to the economy of industry.

## Materials and Methods

### Sample Preparation

**FIGURE 1:**
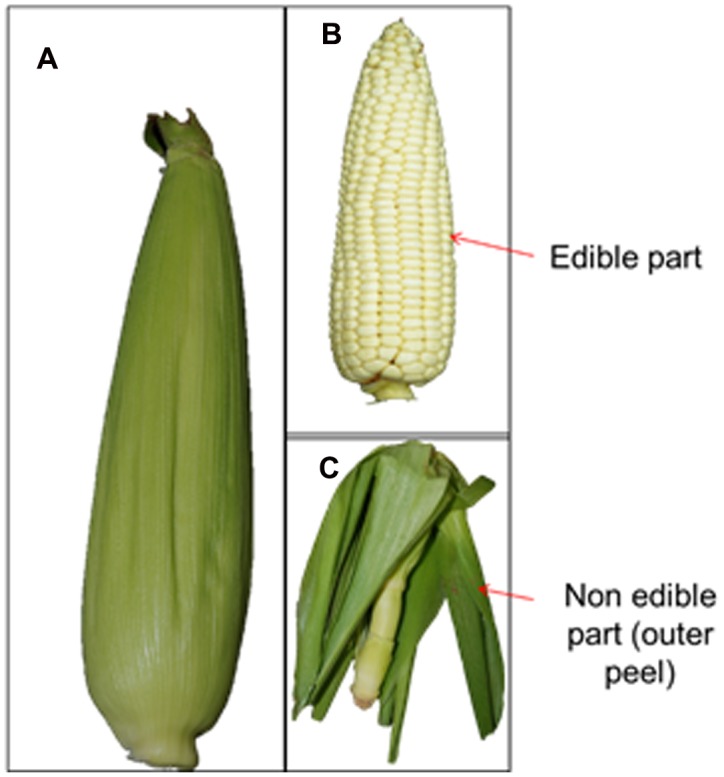
**Fruit (A),** kernel **(B)**, and non-edible leaves **(C)** of maize corn (*Zea mays*).

The whole corn of *Z. mays* L. (**Figure 1A**) was purchased from a local market located at Gyeonsan, Republic of Korea. The ear leaves (**Figure 1C**) were collected from the corn (**Figures 1A,B**) and cut into small pieces of approximately 1 cm. A total of 20 g of leaf pieces were then placed in a 250 mL conical flask, after which 100 mL of double distilled water was added and the samples were boiled for 15 min with continuous stirring. The aqueous extract of corn leaves (ACL) was cooled to room temperature, filtered and stored at 4°C before being used for the synthesis of AgNPs.

### Biosynthesis of AgNPs

The synthesis of AgNPs was conducted by the green synthesis route using ACL. Briefly, 20 mL of ACL was added to 500 mL conical flasks containing 200 mL of 1 mM AgNO_3_ and stirred continuously at room temperature until the solution became reddish brown. The concentration of ACL to AgNO_3_ was maintained at 1:10 ratio with the use of less concentration of ACL and AgNO_3_ in order to control the shape and size of the nanoparticles.

### Characterization of AgNPs

The newly synthesized AgNPs were characterized by UV-VIS spectroscopy, scanning electron microscopy (SEM), energy-dispersive X-ray spectroscopy (EDS), Fourier-transform infrared spectroscopy (FT-IR), thermogravimetric and differential thermogravimetric (TGA/DTG) analysis, and X-ray powder diffraction (XRD) using standard analytical procedures (Basavegowda and Lee, 2013; Ramamurthy et al., 2013).

The synthesis of the AgNPs was monitored by the UV-Vis spectroscopy analysis by measuring the absorption spectra between 350 and 550 nm at a resolution of 1 nm using a microplate reader (Infinite 200 PRO NanoQuant, TECAN, Mannedorf, Switzerland). Changes in the color of the reaction mixture were observed every 3 h during incubation. The surface morphology of the AgNPs was analyzed using FE-SEM. The AgNPs were powdered using an agate mortar and pestle, then uniformly spread over the sample holder and sputter coated with platinum in an ion coater for 120 s, after which they were observed by FE-SEM (S-4200, Hitachi, Japan). Elemental composition analysis of the powdered AgNPs was conducted using an EDS detector (EDS, EDAX Inc., Mahwah, NJ, USA) attached to the FE-SEM machine. FT-IR analysis of the powdered AgNPs and the ACL extract was conducted using a FT-IR spectrophotometer (Jasco 5300, Jasco, Mary’s Court, Easton, MD, USA) in the wavelength range of 400–4000 cm^-1^. The powdered AgNPs sample was blended with potassium bromide (KBr) in a 1:100 ratio using an agate mortar and pestle, then compressed into a 2 mm semi-transparent disk using a specially designed screw knot, after which different modes of vibrations were analyzed for the presence of different types of functional groups in AgNPs and ACL extract.

Effects of high temperature on synthesized AgNPs were evaluated using a TGA machine (SDT Q600, TA Instruments, New Castle, DE, USA). For TGA analysis, powdered AgNPs (3.0 mg) were placed in an alumina pan and heated from 20 to 700°C at a ramping time of 10°C/min under a N_2_ atmosphere in a specially designed heating chamber. The corresponding weight loss data were recorded using a computer attached to the TG/DTG machine with the SDT software. The AgNPs nanoparticles were analyzed by XRD (X’Pert MRD model, PANalytical, Almelo, The Netherlands). Prior to use, the AgNPs were dried at 60°C in a vacuum oven and ground to fine powder using an agate mortar and pestle. The samples were uniformly spread over the glass sample holder and subsequently analyzed at 30 kV and 40 mA with Cu Kα radians at an angle of 2𝜃. The average particle diameter of AgNPs was calculated from the XRD pattern, according to the line width of the maximum intensity reflection peak. The size of the nanoparticles was calculated through the Scherer equation (Yousefzadi et al., 2014).

### Biological Activity of AgNPs

#### Antibacterial Activity of AgNPs

The antibacterial potential of AgNPs was determined against five different foodborne bacteria (*B. cereus* ATCC 13061, *L. monocytogenes* ATCC 19115, *S. aureus* ATCC 49444, *E. coli* ATCC 43890, and *S.* Typhimurium ATCC 43174) by the standard disk diffusion method (Diao et al., 2013). The bacterial pathogens were obtained from the American Type Culture Collection (ATCC, Manassas, VA, USA) and maintained on nutrient agar media (Difco, Becton, Dickinson and Company, Sparks Glencoe, MD, USA). Prior to use, the colloidal solution of the AgNPs was prepared by dissolving AgNPs in 5% dimethyl sulfoxide (DMSO, 1000 μg/mL) and sonicating the samples at 30°C for 15 min. Filter paper disks containing 50 μg of AgNPs/disk were used for the assay. Standard antibiotics, kanamycin and rifampicin, at 5 μg/disk were taken as positive controls, while 5% DMSO was used as the negative control. The overnight grown cultures of tested bacteria were diluted to 1 × 10^-7^ colony forming unit were used for the assay. The antibacterial activity of the AgNPs was determined by measuring the diameter of zones of inhibition after 24 h of incubation at 37°C. The minimum inhibitory concentration (MIC) and minimum bactericidal concentration (MBC) of the AgNPs were determined by the two-fold serial dilution method (Kubo et al., 2004). Different concentrations of AgNPs (100–3.12 μg/mL) were used for MIC test. Prior to test, initially 200 μg of the AgNP was added to initial tube containing, 2 mL of NB media, then 1 mL from it was transferred to next tube which contains 1 mL of only NB media and mixed properly, then the dilution was made till the concentration of the last tube was 3.12 μg/mL. The control tube contains only 1 mL of NB media. Then 10 μL of the tested pathogen was added to each tube. This procedure was repeated for all the tested pathogens. Then all the tubes were mixed properly and were incubated at 37°C overnight in a shaker incubator. The lowest concentration of AgNPs that did not show any visible growth of test organisms was determined as the MIC. Further, the MIC concentration and the next higher concentration were spread on NA plates and incubated for another 24 h at 37°C. The concentration that did not show any growth of a single bacterial colony on the NA plates was defined as the MBC value. Both MIC and MBC values were expressed as μg/mL.

#### Synergistic Potential of AgNPs

The synergistic activity of the AgNPs was determined with antibiotics (kanamycin and rifampicin) or anticandidal agent (amphotericin b).

##### Synergistic antibacterial activity of AgNPs

The synergistic antibacterial potential of AgNPs, as well as kanamycin and rifampicin as a standard antibiotics was determined against five foodborne pathogenic bacteria, *B. cereus* ATCC 13061, *E. coli* ATCC 43890, *L. monocytogenes* ATCC 19115, *S. aureus* ATCC 49444, and *S.* Typhimurium ATCC 43174 by the standard disk diffusion method (Naqvi et al., 2013). The bacterial pathogens were freshly cultured on nutrient broth media (Difco, Becton, Dickinson and Company, Sparks Glencoe, MD, USA). AgNPs (1 mg/mL) and the standard antibiotics (kanamycin or rifampicin at 200 μg/mL) were mixed properly at a 1:1 ratio and sonicated for 15 min at room temperature. Different antibiotic disks were prepared by adding 50 μl of the AgNPs/antibiotics mixture solution to a 6 mm filter paper disk that contains 25 μg AgNPs and 5 μg antibiotics together. The synergistic antibacterial activity of the AgNPs/antibiotics mixture was measured after 24 h of incubation at 37°C in terms of the diameters of the zones of inhibition around the filter paper disks.

##### Synergistic anticandidal activity of AgNPs

The synergistic anticandidal potentials of AgNPs and amphotericin b, a standard antifungal agent, were determined against five different pathogenic *Candida* species, *C. albicans* KACC 30003 and KACC 30062, *C. glabrata* KBNO6P00368, *C. geochares* KACC 30061 and *C. saitoana* KACC 41238, by the disk diffusion method (Murray et al., 1995). These *Candida* species were obtained from the Korean Agricultural Culture Collection (KACC, Suwon, Republic of Korea). AgNPs (2 mg/mL) and amphotericin b (200 μg/mL) were mixed in a 1:1 ratio and sonicated for 15 min at room temperature. Paper disks were prepared by adding 50 μL of the AgNPs/amphotericin b mixture solution to a 6 mm filter paper disk that contains 50 μg AgNPs and 5 μg amphotericin b. The *Candida* species in liquid media were spread uniformly on potato-dextrose agar (PDA) media (Difco, Becton, Dickinson and Company, Sparks Glencoe, MD, USA), after which the anticandidal disks were placed on the plates and samples were incubated at 28°C for 48 h. The synergistic anticandidal activity of the AgNPs/amphotericin b mixture solution was determined by measuring the diameters of the zones of inhibition around the paper disk.

### Antioxidant Activity of AgNPs

The antioxidant potential of the AgNPs was determined by 1,1-diphenyl-2-picrylhydrazyl (DPPH) radical scavenging, nitric oxide (NO) scavenging, 2,2′-azino-bis(3-ethylbenzothiazoline-6-sulphonic acid) (ABTS) radical scavenging and reducing power assays.

#### DPPH Radical Scavenging Activity of AgNPs

The DPPH free radical scavenging potential of AgNPs was determined as previously described (Patra et al., 2015). Briefly, five different concentrations (20–100 μg/mL) of AgNPs and ascorbic acid (ASA) as the standard reference compound was assayed. The absorbance of the reaction mixtures was recorded at 517 nm using the microplate reader and the results were interpreted as the percentage scavenging according to the following equation:

$$\text{Percentage scavenging }=\frac{{\text{Abs}}_{\text{C}}-{\text{Abs}}_{\text{T}}}{{\text{Abs}}_{\text{C}}}\times 100$$

where, Abs*_C_* is the absorbance of the control and Abs*_T_* is the absorbance of the treatment.

#### NO Scavenging Activity of AgNPs

The NO scavenging potential of AgNPs was determined by the standard procedure (Makhija et al., 2011). Five different concentrations (20–100 μg/mL) of AgNPs and ASA as the standard reference compound were taken for the assay. The absorbance of the reaction mixtures was recorded at 546 nm using a microplate reader, after which the results were calculated as the percentage scavenging activity according to Eq. 1.

#### ABTS Radical Scavenging Activity of AgNPs

The ABTS radical scavenging potential of AgNPs was determined by the standard procedure (Thaipong et al., 2006). Briefly, five different concentrations (20–100 μg/mL) of AgNPs and ASA as the standard reference compound was taken for the assay. The absorbance of the reaction mixtures was recorded at 750 nm using a microplate reader, and the results were interpreted according to Eq. 1.

#### Reducing Power of AgNPs

The reducing power of AgNPs was determined by the standard procedure (Sun et al., 2011). Briefly, five different concentrations (20–100 μg/mL) of AgNPs and ASA as the standard reference compound were assayed. The absorbance of the reaction mixture was measured at 700 nm against an appropriate control and the results were expressed as OD values at 700 nm.

### Statistical Analysis

The results of all the experiments were expressed as the mean value of three independent replicates ± the standard deviation (SD). Statistical analysis of the significance differences between the mean values of the results were identified by one-way analysis of variance (ANOVA) followed by Duncan’s test at the 5% level of significance (*P* < 0.05) using the Statistical Analysis Software (SAS) (Version: SAS 9.4, SAS Institute Inc., Cary, NC, USA).

## Results

### Synthesis of AgNPs

The industrial wastes from maize plants (**Figures 1A,C**) after utilization of the kernels (**Figure 1B**) were used in the present study for synthesis of AgNPs. Biosynthesis of AgNPs was indicated by gradual color development in the reaction solution after 1 h of incubation and subsequent increases in the intensity of the color during the course of reaction. The formation of AgNPs was monitored by a number of characterization techniques as described below.

### Characterization of AgNPs

**FIGURE 2:**
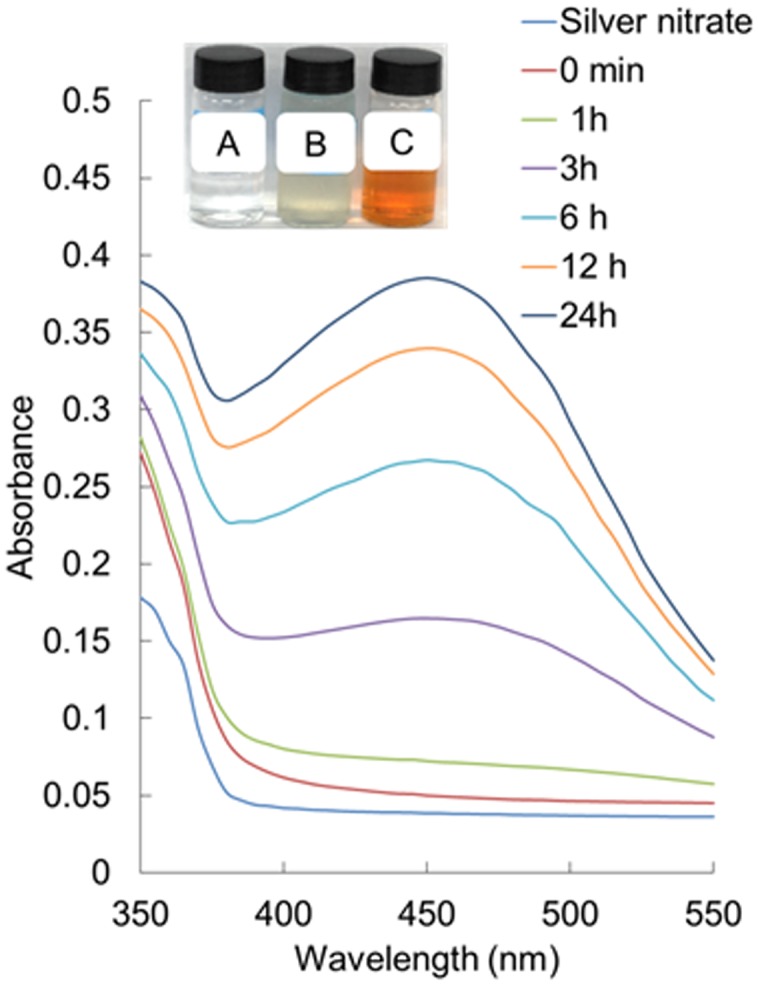
**UV-visible spectra of silver nanoparticles (AgNPs) synthesized by the aqueous corn leaves extracts (ACL)**. Inset: change in color of the solution confirming the synthesis of AgNPs (A – AgNO_3_ solution, B – aqueous corn leaves extracts (ACL), and C – AgNPs).

The UV-Vis spectra of the synthesized AgNPs recorded at different time intervals are presented in **Figure 2**. The absorbance peaks indexed as different colors indicated the reduction of AgNO_3_ by ACL with different time intervals (0 min, 30 min, 1 h, 3 h, 6 h, 12 h, and 24 h) at room temperature (**Figure 2**). The UV-Vis spectra of the synthesized AgNPs were further recorded after 24 h, but the intensity of the color did not intensify after 24 h, confirming that the reaction was completed within 24 h.

**FIGURE 3:**
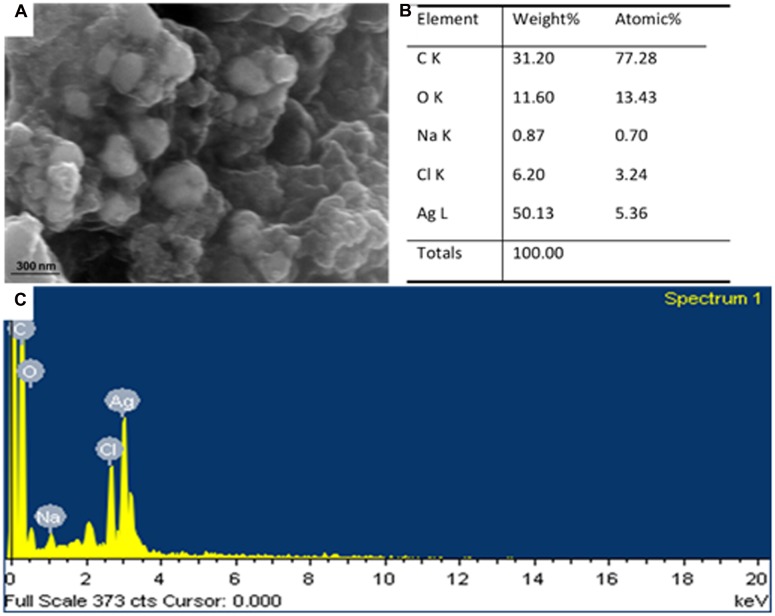
**Scanning electron microscopy image** **(A)** and energy-dispersive X-ray analysis **(B,C)** of silver nanoparticles (AgNPs) synthesized by the aqueous extracts of corn leaves (ACL).

**FIGURE 4:**
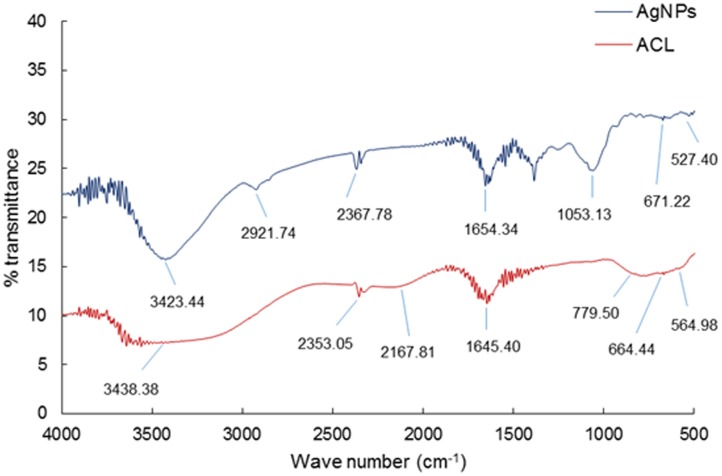
**Fourier-transformed infrared spectroscopy analysis of silver nanoparticles (AgNPs) and the aqueous extracts of corn leaves (ACL)**.

The morphology of the synthesized AgNPs was revealed by FE-SEM analysis (**Figure 3A**). The FE-SEM image revealed the formation of a cluster of spherical beadlike structures of AgNPs that were strongly aggregated. The elemental composition of the synthesized AgNPs was determined by an EDS machine attached to the FE-SEM. The elemental composition confirmed that AgNPs were composed of 50.13% Ag, 31.20% C, 11.60% O, 6.20% Cl, and 0.87% Na (**Figures 3B,C**). FT-IR analysis of the ACL and AgNPs is shown in **Figure 4**. Absorption peaks located at 3438.38, 2353.05, 2167.81, 1645.40, 779.50, 664.44, and 564.98 cm^-1^ were observed upon ACL, whereas absorption peaks located at 3423.44, 2921.74, 2367.78, 1654.34, 1053.13, 671.22, and 527.40 cm^-1^ were observed for the AgNPs (**Figure 4**).

**FIGURE 5:**
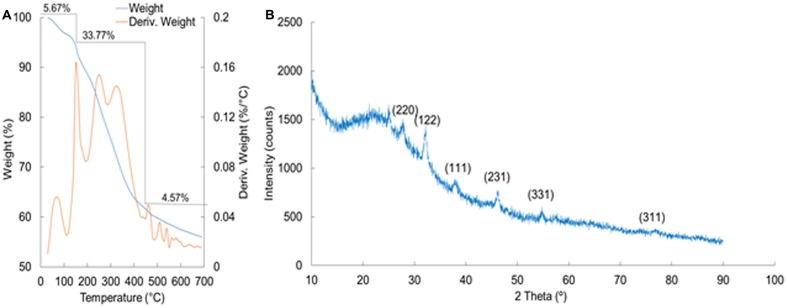
**Thermogravimetric and differential thermogravimetric (TG/DTG) analysis (A)** and X-ray diffraction analysis **(B)** of silver nanoparticles (PE-AgNPs) synthesized by the aqueous extracts of corn leaves (ACL).

Thermogravimetric and differential thermogravimetric analysis of the synthesized AgNPs was conducted to show the nature of AgNPs at higher temperature (**Figure 5A**). A total of 44.01% weight loss was observed in three different phases when the AgNPs were heated to 700°C in a controlled N_2_ atmosphere. The first phase of weight loss was observed between 30 and 150°C with a weight loss of 5.67%. In this phase, the water molecules that were attached to the AgNPs during the course of synthesis were degraded. The second phase of weight loss was observed between 150 and 470°C with a maximum weight loss of 33.77%. During this phase, organic molecules, such as alkanes, phenols, alkenes, proteins, and polysaccharides from the ACL that contributed to the reduction of the AgNPs as capping and stabilizing agents were degraded. The third phase extended from 480 to 700°C, during which time there was a weight loss of 4.57%. The nature of the synthesized AgNPs was analyzed by XRD (**Figure 5B**). The diffraction pattern showed six diffraction peaks at 27.78°, 32.04°, 38.41°, 46.12°, 54.95°, and 76.78°, which corresponded to (220), (122), (111), (231), (331), and (311) planes of silver, respectively. The average crystal size of the silver crystallites was calculated from the full width at half maximum (FWHMs) values of the diffraction peaks, using the Scherer equation. The estimated size of crystallite in different planes of silver was determined as 31.18, 35.74, and 69.14 nm with the mean value of all three peaks as 45.26 nm.

### Biological Activity of AgNPs

#### Antibacterial Activity of AgNPs

**FIGURE 6:**
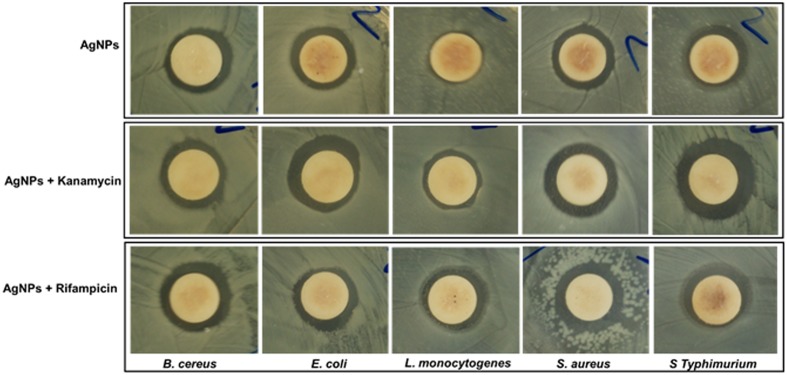
**Antibacterial activity of AgNPs (50 μg/disk) and synergistic antibacterial potential of AgNPs (25 μg) mixed with standard antibiotics, kanamycin (5 μg), and rifampicin (5 μg)**.

The AgNPs at 50 μg/disk displayed moderate antibacterial activity against all five foodborne pathogenic bacteria, as indicated by diameter of inhibition zones of 9.26–11.57 mm (**Table 1**; **Figure 6**). The standard antibiotics, kanamycin, and rifampicin, at 5 μg/disk did not show any inhibitory activity against any of the five pathogens. Among the pathogenic bacteria, AgNPs were more active against *S. aureus* (11.57 mm inhibition zone) than *L. monocytogenes* (9.26 mm inhibition zone). The MIC and the MBC values of AgNPs against all five pathogenic bacteria ranged from 12.5 to 100 μg/mL (**Table 1**).

**Table 1:** Antibacterial activity of AgNPs against five foodborne pathogenic bacteria.

Bacteria	AgNPs (50 μg/disk)	MIC (μg/mL)	MBC (μg/mL)
*B. cereus* ATCC 13061	11.39 ± 1.2^a∗^	25	50
*E. coli* ATCC 43890	10.55 ± 0.27^b^	50	100
*L. monocytogenes* ATCC 19115	9.26 ± 0.31^c^	25	50
*S. aureus* ATCC 49444	11.57 ± 0.25^a^	12.5	25
*S.* Typhimurium ATCC 43174	11.22 ± 0.38^a^	50	100

*^∗^Data are expressed as the mean zone of inhibition in mm ± SD. Values with different superscript letters are significantly different at *P* < 0.05*.

#### Synergistic Antimicrobial Potentials of AgNPs

##### Synergistic antibacterial potential of AgNPs

The synergistic potential of the AgNPs together with the standard antibiotics, kanamycin and rifampicin, were evaluated against all five foodborne pathogenic bacteria and the results are presented in **Table 2** and **Figure 6**. At low concentrations (5 μg/disk), neither antibiotics exhibited any positive activity against any of the five pathogenic bacteria, which was also true for AgNPs at 25 μg/disk concentration. Thus, to study the synergistic antibacterial potential, both antibiotics and AgNPs were combined at this low concentration and their activities were tested against the five foodborne pathogens. When both antibiotic and AgNPs were mixed, they displayed strong antibacterial activity against all pathogens, with zones of inhibition ranging in diameter from 10.62 to 14.33 mm (**Table 2**).

**Table 2:** Synergistic antibacterial activity of AgNPs (25 μg) with standard antibiotics, kanamycin (5 μg) or rifampicin (5 μg), against foodborne pathogenic bacteria.

Bacteria	AgNPs + Kanamycin	AgNPs + Rifampicin
*B. cereus* ATCC 13061	11.67 ± 0.16^c∗^	12.76 ± 0.44^b^
*E. coli* ATCC 43890	12.45 ± 2.02^b^	11.43 ± 0.21^c^
*L. monocytogenes* ATCC 19115	10.62 ± 0.29^d^	11.64 ± 0.23^c^
*S. aureus* ATCC 49444	12.57 ± 0.20^b^	14.33 ± 0.40^a^
*S.* Typhimurium ATCC 43174	12.80 ± 0.31^a^	11.45 ± 0.19^c^

*^∗^Data are expressed as the mean zone of inhibition in mm ± SD. Values in each same column with different superscript letters are significantly different at *P* < 0.05*.

##### Synergistic anticandidal potential of AgNPs

**FIGURE 7:**
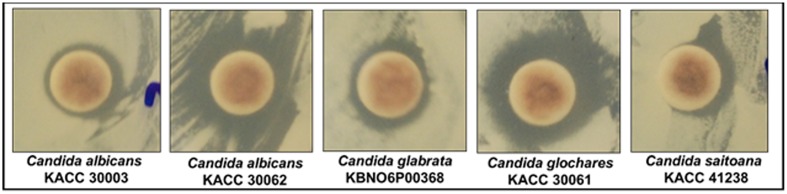
**Synergistic anticandidal potential of AgNPs (50 μg) mixed with a standard amphotericin b (5 μg)**.

The synergistic anticandidal activities of the AgNPs are presented in **Table 3** and **Figure 7**. AgNPs at a concentration of 50 μg/mL did not exhibit any anticandidal activity against the five tested *Candida* species. However, when AgNPs (50 μg/disk) were combined with a standard anticandidal agent, amphotericin b (5 μg/disk), they displayed potent anticandidal activity against all five *Candida* species, with zones of inhibition ranging from 9.74 to 14.75 mm (**Table 3**; **Figure 7**).

**Table 3:** Synergistic anticandidal activity of AgNPs (50 μg) with a standard antifungal agent, amphotericin b (5 μg), against pathogenic *Candida* species.

*Candida* species	Mean inhibition zone in mm ±*SD*
*C. albicans* KACC 30003	10.34 ± 0.29^cd^∗^^
*C. albicans* KACC 30062	12.88 ± 0.15^b^
*C. glabrata* KBNO6P00368	10.98 ± 0.71^c^
*C. glochares* KACC 30061	14.75 ± 0.30^a^
*C. saitoana* KACC 41238	9.74 ± 0.14^d^

*^∗^Values with different superscript letters are significantly different at *P* < 0.05*.

#### Antioxidant Activity of AgNPs

**FIGURE 8:**
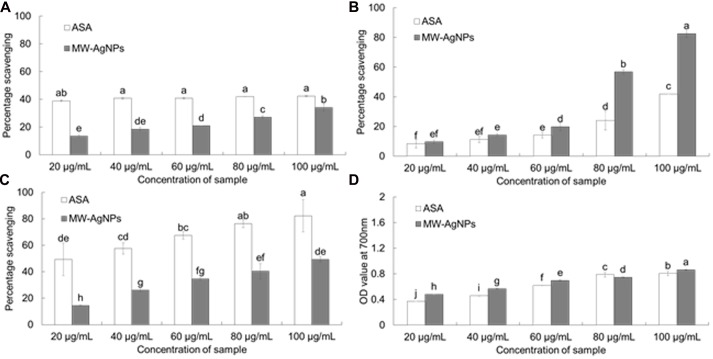
**Antioxidant potentials of AgNPs and ascorbic acid (ASA). (A)** DPPH radical scavenging. **(B)** Nitric oxide scavenging. **(C)** ABTS radical scavenging. **(D)** Reducing power assay. Different superscript letters in each column indicate significant differences at *P* < 0.05.

The antioxidant potential of synthesized AgNPs was determined by *in vitro* assays of DPPH radical scavenging, NO scavenging, ABTS radical scavenging and reducing power. The DPPH radical scavenging potential of AgNPs is presented in **Figure 8A**. AgNPs displayed a moderate DPPH radical scavenging potential of 34.09% at 100 μg/mL, whereas ASA, which was taken as the reference standard, showed comparably high DPPH scavenging activity of 42.41% at 100 μg/mL (**Figure 8A**). AgNPs exerted comparably high NO scavenging potential of 82.63% at 100 μg/mL compared with that of 41.95% of ASA at 100 μg/mL (**Figure 8B**). The ABTS radical scavenging potential of AgNPs is presented in **Figure 8C**. AgNPs exhibited a moderate value of 49.29% ABTS radical scavenging potential relative to 82.20% by ASA at 100 μg/mL. AgNPs also displayed strong reducing power (**Figure 8D**).

## Discussion

The concentration of ACL to AgNO_3_ was maintained at 1:10 ratio with the use of less concentration of ACL and AgNO_3_ in order to obtain small and controlled size of nanoparticles. It is presumed that less the concentration of the plant extracts and AgNO_3_ used, the smaller will be the size of the nanoparticles. This hypothesis has been proved by several researchers (Ghosh et al., 2011; Chandran et al., 2014) and can be confirmed again by the present study. The appearance of brown color in the reaction solution (**Figure 2**, inset) was a clear indication of the formation of AgNPs in the reaction mixture (Wu et al., 2004; Kumar et al., 2008; Kumari and Philip, 2013). The characterization of the synthesized AgNPs was achieved using techniques, such as UV-Vis spectroscopy, FE-SEM, EDS, FT-IR, TG/DTG analysis, and XRD analysis (Zhang et al., 2006; Choi et al., 2007; Vilchis-Nestor et al., 2008). These techniques are used for determination of different parameters, such as nature, particle size, characteristics, crystallinity, and surface area.

Spectral analysis revealed that the surface plasmon resonance phenomena (SPR) absorption maxima peak of the synthesized AgNPs occurred at 450 nm with a high absorbance value specific for AgNPs (**Figure 2**) (Kelly et al., 2003; Nazeruddin et al., 2014). In general, typical AgNPs show characteristic SPR at wavelengths ranging from 400 to 480 nm (Pal et al., 2007; Nazeruddin et al., 2014), which was also observed in the present investigation. The SPR absorbance is sensitive to the shape, size and nature of particles present in the solution, and also depends upon inner particle distance and the surrounding media (Nazeruddin et al., 2014). The surface morphology was seen by FE-SEM (**Figure 3A**) and its elemental composition by EDS analysis (**Figures 3B,C**). The EDS pattern indicates that the synthesized AgNPs were crystalline in nature, which is caused by the reduction of silver ions. A strong typical absorption peak was observed at 3.0 keV, which is typical of the absorption of metallic silver nanocrystallites due to SPR (Das et al., 2013) (**Figure 3C**). Similar results upon SEM and EDS analysis of different types of AgNPs were reported previously (Vijaykumar et al., 2013; Nazeruddin et al., 2014; Muthukrishnan et al., 2015; Velusamy et al., 2015).

The intense peaks in FT-IR spectra of both ACL and AgNPs (**Figure 4**) located at 3438 and 3423 cm^-1^ corresponded to O–H stretching of the alcohols and phenolic compounds, while the intense peaks at 1654 and 1645 cm^-1^ corresponded to – C=C-H stretching of the alkenes group (Ramamurthy et al., 2013; Rajeshkumar and Malarkodi, 2014). A major peak was observed at 2921 cm^-1^ in AgNPs, which could be assigned to the C-H stretching vibrations of methyl, methylene, and methoxy groups (Feng et al., 2009). The mechanism of adsorption and capping of AgNPs by ACL can be explained through the coordination of carbonyl bonds (3423 cm^-1^) and subsequent electron transfer from C=O to AgNPs (Qiu et al., 2006). The peak at 3423 cm^-1^ that corresponds to O-H stretching, 1654 cm^-1^ that corresponds to – C=C-H stretching of the alkenes group, and 1053 cm^-1^ might be contributed to by the C-O groups of the polysaccharides in the ACL extract that acted as reducing, capping, and stabilizing agents for the synthesis of AgNPs (Muthukrishnan et al., 2015). The slight shifting in the position of different peaks in the AgNPs from the ACL extract might have been due to progression of the reduction reaction with capping and stabilization of AgNPs by the various secondary metabolites present in the ACL. It is thus evident from the FT-IT spectra of the AgNPs that the bioactive compounds such are polyphenols (chlorogenic acid, caffeic acid, rutin, ferulic acid, morin, quercetin, naringenin, and kaempferol), anthocyanins, flavonoids, flavonols, and flavanols which were previously reported to be present in *Z. mays* (Ramos-Escudero et al., 2012; Bacchetti et al., 2013; Pandey et al., 2013) plays a vital role in the capping and stabilization of the AgNPs.

Thermogravimetric analysis of the AgNPs during this period (**Figure 5A**) indicated that the organic molecules from the ACL had mostly taken part in the synthesis and capping of the AgNPs, but were degraded at higher temperatures (Shaik et al., 2013). The nature of the synthesized AgNPs analyzed by XRD showed that the six diffraction peaks corresponded to (220), (122), (111), (231), (331), and (311) planes of silver, respectively, as per the standard FCC structures of Ag (JCPDS Card no. 04-0783) (Khurana et al., 2014; Roy et al., 2015). This structural characteristic pattern confirmed that the AgNPs had a crystalline structure. (**Figure 5B**).

The development of resistant pathogenic strains has recently affected healthcare systems worldwide (Rajeshkumar and Malarkodi, 2014). Therefore, the positive effects of AgNPs toward a number of foodborne pathogenic bacteria (**Table 1**) could be useful in formulation of new antibacterial drugs against resistant bacterial pathogens. Silver exhibits toxicity toward microorganisms but little or no toxicity toward animal cells; therefore, these properties of Ag particles could be more beneficial for AgNPs in the development of more potent drugs against the pathogens.

The exact cause of the antibacterial action of AgNPs against the pathogenic bacteria is not completely understood. Few studies have shown that the electrostatic attraction between negatively charged bacterial cells and the positively charged nanoparticles could be responsible for its bactericidal effects (Sondi and Salopek-Sondi, 2004). There are several possible proposed mechanisms for the positive antibacterial activity of AgNPs which includes the degradation of enzymes, inactivation of cellular proteins and breakage of DNA (Jiang et al., 2004; Sharma et al., 2009; Guzman et al., 2012). It is presumed that due to smaller size the AgNPs might have attached to the surface of the bacterial cell membrane and disturbed its power functions, such as permeability and respiration and then it could have easily penetrate to the inside of the bacteria and could have caused further damage, possibly by interacting with sulfur- and phosphorus-containing compounds, such as DNA resulting in cell lysis (Gibbins and Warner, 2005; Guzman et al., 2012; Singh et al., 2014; Yousefzadi et al., 2014; Ramesh et al., 2015; Swamy et al., 2015). Ovington (2004) reported the potential antimicrobial activity of nanocrystalline silver products by the process of releasing a cluster of highly reactive silver cations and radicals inside the pathogen body or the cell surface, this could be a possible reason for the antibacterial activity of AgNPs in the present study. The main possible mechanism of antimicrobial action of AgNPs could be that, due to the dissolution of AgNPs, antimicrobial Ag^+^ ions are released which can interact with sulfur-containing proteins in the bacterial cell wall, which may lead to compromised functionality (Levy, 1998; Lansdown, 2004; Ovington, 2004; Reidy et al., 2013).

Similarly, many authors have also reported that due to the smaller size, the interaction of the nanoparticles is better with the targeted pathogen and thus these were more effective (Panacek et al., 2006; Suriya et al., 2012). In addition to this, it is also believed that the AgNPs after penetration into the bacteria membrane might have inactivated their enzymes, generating hydrogen peroxide that ultimately resulted in the death of the bacteria (Raffi et al., 2008). Furthermore, it is also believed that the high affinity of silver for sulfur or phosphorus compounds could be a possible reason for its antibacterial activity against the pathogenic bacteria because as sulfur and phosphorus are abundantly found throughout the cell membrane, the AgNPs could have reacted with sulfur-containing proteins inside or outside the cell membrane and in turn affected the cell viability causing leakage of bacteria leading to lysis (Hamouda and Baker, 2000). Morones et al. (2005) have reported the presence of AgNPs not only at the surface of cell membrane, but also inside the bacteria using the scanning tunneling electron microscopy (STEM), this proves that due to their smaller size, AgNPs have penetrated inside the bacteria and fungi, causing damage by interacting with phosphorus- and sulfur-containing compounds such as DNA. It is evident that with the decrease in the size of the particles to the nanoscale range, the specific surface area of a dose of nanoparticles increases, which allows for greater material interaction with the surrounding environment such as the cell membrane of the targeted pathogenic bacteria. Thus for the inherently antibacterial materials, such as zinc and silver, increasing the surface to volume ratio enhances the antibacterial effect that results in positive antimicrobial activity due to a number of reasons, such as the release of antibacterial metal ions from the particle surface and the antibacterial physical properties of a nanoparticle related to cell wall penetration or membrane damage (Seil and Webster, 2012). It has also been reported that the crystallographic structure surface and high surface-to-volume ratio increase the contact area of metallic nanoparticles with the body of the microorganism that influences the antibacterial activity of nanosized silver particle (Kora and Arunachalam, 2011). These properties of AgNPs made it a potential candidate for the industries in development of modern antimicrobial products. Moreover, the AgNPs could be useful in the formulation of polymer materials for packaging of food items and other durable materials that could be affected by microorganisms (Rhim and Ng, 2007; Finnigan, 2009).

Further study on the synergistic antibacterial activity of AgNPs with the antibiotics that showed as strong positive result (**Table 2**) could be due to the easy penetration of the mixture solution into the bacterial cell membrane, causing serious damage to the cells and death of the bacteria. This synergistic potential of AgNPs with the antibiotics could help minimize the extensive use of antibiotics that has resulted in development of many antibiotic resistant strains. Previously, the positive synergism impact of nanoparticle-antibiotics combination at significantly low concentration have been demonstrated against a number of dreadful multi-drug resistant pathogenic bacteria (Li et al., 2005; Birla et al., 2009; Ruden et al., 2009; Fayaz et al., 2010; Hwang et al., 2012; McShan et al., 2015; Barapatre et al., 2016; Deng et al., 2016; Panacek et al., 2016). All these authors have proposed that such positive results of nanoparticle-antibiotics combination might be due to the differences in size of prepared Ag-NPs the bonding reaction between them which enable the mixture to better interact with the pathogen.

Similarly, the synergistic anticandidal activities of the AgNPs as presented in **Table 3** and **Figure 7** confirmed that the use of AgNPs together with lower concentrations of anticandidal agents could be beneficial in clinical applications by enabling the application of lower amounts of anticandidal agents to avoid adverse effects and development of drug resistant pathogens (Gajbhiye et al., 2009). Candida are among the most common pathogenic yeast influencing humans, but treatments for Candida infections are limited because of development of resistant *Candida* sp., limited availability of antifungal drugs and high costs (Khan et al., 2003). Thus, the use of AgNPs together with low concentrations of amphotericin b has the potential for improved treatment of Candida related diseases. There have been only several reports on the antibacterial effect of AgNPs against *Candida* sp. (Panacek et al., 2009; Vazquez-Munoz et al., 2014; Artunduaga Bonilla et al., 2015; Lara et al., 2015), and the present study also corroborates with previous claim. As a previous report of the effective synergistic effects of AgNPs combined with antibiotics (Monteiro et al., 2013), the present study also corroborates with their findings.

The strong antioxidant potential of the AgNPs (**Figure 8**), could also make it a good source of natural agent for antioxidant. The strong DPPH and NO scavenging potential of AgNPs could make it a potential candidate for drug delivery. The moderate ABTS scavenging potential of AgNPs compared to ASA might be due to the different types of functional groups from the ACL that have attached to the surface of AgNPs during synthesis and capping of AgNPs (Adedapo et al., 2008). AgNPs also displayed strong reducing power, which might be attributed to the presence of phenolic compounds from ACL extract on the surface of AgNPs as surface stabilizers and capping agents. It is observed in the present investigation that the antioxidant activity of the AgNPs was higher than that of the standard ASA which might be possible due to the size and crystalline nature of the AgNPs as reported by various authors (El-Rafie and Abdel-Aziz Hamed, 2014; Bhakya et al., 2016; Gopukumar et al., 2016).

## Conclusion

In the present study, AgNPs were synthesized using the aqueous extract of *Z. mays* corn leaves, which is a novel approach of waste utilization in nanoparticle synthesis. The results revealed that the synthesized nanoparticles are within the nanometer range, with an SPR of 450 nm based on UV-Vis spectroscopy. The elemental composition and crystallinity structure confirmed the synthesized particles to be Ag. The AgNPs displayed positive antibacterial activity against different foodborne pathogenic bacteria, as well as strong synergistic antibacterial and anticandidal activity with low concentrations of antibiotics and anticandidal agents. The AgNPs exhibited strong antioxidant potential. Based on these results, this approach of utilization of industrial wastes in nanoparticle synthesis can be beneficial in large scale fabrication of nanomaterials. The synergistic study of AgNPs with common antibiotics and anticandidal agents could be beneficial in formulation of antibacterial products and anticandidal drugs to be used in various food, agricultural, cosmetic, and pharmaceutical industries and future platforms for preparing nano-medicines, and targeted drug delivery.

## Author Contributions

JP carried out all the experiment and wrote the manuscript. JP and K-HB designed and edited the manuscript.

## Conflict of Interest Statement

The authors declare that the research was conducted in the absence of any commercial or financial relationships that could be construed as a potential conflict of interest.
